# Social Deprivation and Ethnicity Are Associated with More Problematic Sleep in Middle-Aged and Older Adults

**DOI:** 10.3390/clockssleep5030030

**Published:** 2023-08-07

**Authors:** John A. Groeger, Piril Hepsomali

**Affiliations:** 1 Department of Psychology, School of Social Sciences, Nottingham Trent University, Nottingham NG1 5LT, UK; 2 School of Psychology, University of Roehampton, London SW15 4JD, UK

**Keywords:** sleep quality, sleep duration, sleep problems, age, demographics, sex, race/ethnicity, intersectionality

## Abstract

**Objectives:** We test the hypothesis that the incidence of sleep problems is influenced by socio-demographic variables, particularly social deprivation and ethnicity. **Methods:** Self-reports of sleep duration and sleep difficulties (waking in the night, sleeping in the day, difficulty waking and snoring), personal wealth (household income, property-owning, etc.), ethnic group, employment, education, as well as post-code-based Townsend Social Deprivation, were extracted from UK Biobank’s cohort of c500,000 British-domiciled adults (40–70 years). Analyses contrasted the incidence of different sleep problems and a composite measure of these (the Problematic Sleep Index) across groups. **Results:** Almost one-third of participants reported sleeping shorter (24.7%), or longer (7.7%) than age-corrected recommended sleep durations. The incidence of shorter or longer sleep increased with social deprivation and varied with ethnicity. Snoring, waking during the night, finding it difficult to get up in the morning and sleeping in the daytime were subject to similar effects. The Problematic Sleep Index showed being younger, male, employed, home-owning, having a higher household income, having a higher level of educational achievement, and time in education were all associated with better sleep, as was living in a more affluent area and being White. **Conclusions:** Sleep problems in Britain show a social gradient, independently of a range of other demographic and social influences, suggesting that sleep quality differs with and between ethnic groups. These sleep inequalities suggest that the protective and recuperative effects of sleep are disproportionately distributed across society and should encourage us to consider the potential benefits of community-specific sleep interventions.

## 1. Introduction

Adult sleep reduces in length and undergoes profound structural changes, including reduced slow-wave sleep and increased nocturnal wakefulness, as age increases [1,2]. Although the amount of sleep required to maintain functioning also reduces with age [3], this is at odds with the age-related incidence of sleep complaints [4,5]. There are clear health consequences of inadequate sleep, and health is profoundly influenced by social inequalities. Meta-analyses suggest that sleep problems are also subject to a ‘social gradient’ [6]. Although such integrative reviews are useful, the studies on which they rely have sample sizes that are generally too small to simultaneously quantify the independent effects of the many potential contributors to social inequality, they are often concentrated on particular cities or regions and only the major ethnicities living there, and they vary unhelpfully in terms of what is regarded as adequate sleep. This study addresses these inadequacies by using agreed, age-sensitive criteria for sleep duration, formal definitions of social deprivation and self-declared ethnicity from a large (c500,000) national sample of middle-aged and older adults. Furthermore, we extend the focus of sleep beyond duration to include the various sleep problems individuals report, combining these into a simple, but we hope informative, measure of overall sleep quality.

There is no simple dose-response relationship between sleep duration and mortality or morbidity. Recent meta-analyses show that short [7] and long [8] sleep durations are both associated with an increased risk of mortality. Morbidity shows similar relationships between sleep extremes and diabetes mellitus, cardiovascular, and coronary heart diseases. Other conditions show an increased risk at only one end of the duration continuum: increased risk of stroke and obesity with long sleep [8] and increased incident hypertension with short sleep [7]. Sleeping too much or too little has clear health consequences, but it is unclear what the length of ‘short’ or ‘long’ sleep actually is.

A “short” duration is often, but not always, defined as six hours or fewer, whereas durations longer than nine hours are classed as “long” [9,10,11]. Differences between studies in criteria for adequate sleep duration complicate interpretation, as does the frequent failure to consider the sleeper’s age. According to geographical, sex, and age-representative data for the UK, the lower bound encompasses some 25% of all middle-aged and elderly adults, but at most, 5% exceed the cut-off for longer sleep. In contrast, younger adults’ sleep durations are skewed in the opposite direction [12]. Here we follow expert consensus which has agreed age-specific criteria for excessively short or long sleep [13,14] (Sleep duration cut-offs for younger (Y: 25–64 years old) and older (O: 65 and older) adults, for Short (Y: 6 h; O: 5–6 h) Long (Y:10 h; O: 9 h), and excessively short (Y: <6 h; O: <5 h) and excessively long (Y: =>10 h; O: =>9 h)), but note several shortcomings in the criteria: objectively measured sleep is almost certainly what is important, discrepancies between weekday and weekend sleep are typical of working age adults [15], and it is unlikely that men and women require similar amounts of sleep, given the hormonal differences across menstruation and menopause [16]. Finally, and more fundamentally, it is very unlikely that duration per se is what is important about sleep, but its quality. While there is, of course, a widely used measure of sleep quality [17], achieving expert consensus for what characterised sleep quality has proven more elusive than for sleep quantity [18]. We propose that the absence of sleep complaints is a useful proxy for sleep quality and develop a method for combining a range of complaints into a single measure, capturing complaints typically made about sleep, such as remaining asleep once sleep is initiated, waking early or later than desired, snoring, etc. [12] and some symptoms typical of insomnia identified in DSM-5 and ICSD-3 [19].

Despite some clarion calls for such research [20], remarkably few studies have compared sleep across different ethnic groups, social strata, or geographies. While US studies consistently report that Black/African Americans have more sleep challenges than their White counterparts (see meta-analyses [20,21], such conclusions can obscure the complexity of the issues involved. An empirical study of c500 middle-aged Chicago residents found that objectively measured duration was lower in ‘Black’, ‘Asian’, and ‘Hispanic’ than in ‘White’ participants, having controlled for age, sex, education, work schedule (i.e., day vs. night shift) and a number of medical conditions [22]. A similarly sized Wisconsin-based study of middle-aged and elderly supports these findings but shows that the time awake after sleep onset (WASO) was also longer among Black participants. Importantly, WASO was significantly associated with the level of disadvantage of the areas in which participants lived, but the racial difference remained statistically significant after adjusting for domiciliary disadvantage [23]. Such studies of particular locales are important but understate the complex “intersectionality” of sleep.

Meta-analyses of sleep-related data from larger studies show similar disparities [20,21], but generally, the sample sizes are insufficiently large to unconfound the many socio-demographic differences which may influence these findings. The complexity of the effects of race and social disadvantage is clear from the US National Health Interview Survey (N = 175,244) [24]. Prevalence of “short” sleep durations (i.e., <7 h) was highest among Black respondents in all occupational groups, irrespective of country of birth. Furthermore, the prevalence of these shorter sleep durations increased with occupational status in Black and ‘Latino’ respondents, while it decreased among White respondents. A similar racial difference in self-reported sleep duration occurs in another population-based study (N = 32,749) [25]. ‘Black’ respondents were more likely to report “Short” (≤6 h) and “Long” Sleeping (≥9 h) than sleep durations between these ranges, compared with ‘White’, ‘Mexican American’, ‘Other Hispanic’ and ‘Non-Hispanic’ participants, even after adjustment to control for a wide range of socio-demographic influences. The Ontario Health Study (N = 143,307) [26] reports sleep duration across a wider range of ethnicities, showing that ‘White’ respondents actually slept longer than any group, even though short (<7 h) and long (>9 h) sleepers were more likely self-describe as ‘Aboriginal’/‘Black’/‘Korean’/‘South Asian’/‘West Asian’/‘Mixed’ ethnicity than to be ‘White’. Participants self-describing as ‘Arab’/‘Chinese’/‘Filipino’/‘Japanese’/‘Southeast Asian’ were more likely than ‘White’ participants to be short sleepers but were not more likely to report being long sleepers. While these outcomes hold after adjustments for sex, nativity, and years resident in Canada, the admirably diverse sample and broad age range (18+ yrs; Median 47 yrs) inevitably restrict sample sizes for some analyses. Nor do the data reflect the demography of Ontario, let alone Canada as a whole. While these adjusted-model studies ensure that any racial differences in sleep duration are not a reflection of particular confounds, it does not identify their relative contributions to explaining sleep disparities.

Thus, there is evidence from both self-report and objective sleep measurement that sleep duration, and to a lesser extent, some sleep problems, are affected by race and social deprivation. However, there are few data available for societies outside North America, and thus, it is unclear whether the findings reported above are peculiar to that continent and its pattern of immigration and settlement. Here we report both agreement with simple self-reported questions and a more complex combination of these, based on a very large regionally diverse national sample: the UK Biobank (UKB). We quantify sleep duration in terms of expert consensus regarding what age-specific durations are recommended, or otherwise, we quantify race and ethnicity based on self-chosen, nationally recognised adjectival descriptors, and we distinguish between personal wealth and the level of deprivation prevailing in the area in which the respondent lived, as derived from census-based information (i.e., Townsend Deprivation Index [27]). Our aim is to identify the independent effects of different aspects of participants’ socio-demography on the problems they report about their sleep.

## 2. Results

The average reported sleep duration was 7 h 9 min ± 1 h 7 min (Range 1–23 h; N = 498,289). This is consistent with the average reported from a nationally representative face-to-face survey (7 h 2 min ± 1 h 33 min) [12]. In terms of the various cut-offs for short and long sleep duration used in previous studies mentioned above, 5.5% of respondents claimed to sleep for five hours or less, 24.7% for six hours or less, 63.3% reported sleeping for seven hours or less, while 7.7% reported sleeping for more than eight hours, and 1.9% reported sleeping for nine or more hours per night.

### 2.1. Inadequacy of Sleep Duration

Social deprivation, as quantified by the Townsend Deprivation Index for respondents’ domiciliary address, was strongly associated with ethnicity (χ^2^_(12)_ = 20,559.20, *p* < 0.0001; n = 497,672; ϕ = 0.204; see Table 1). White participants were much more likely to be domiciled in affluent areas, and Black participants were more likely to be living in deprived areas. Ethnicities broadly summarised as Asian and Mixed were also more likely to live in affluent areas.

**Figure 1:**
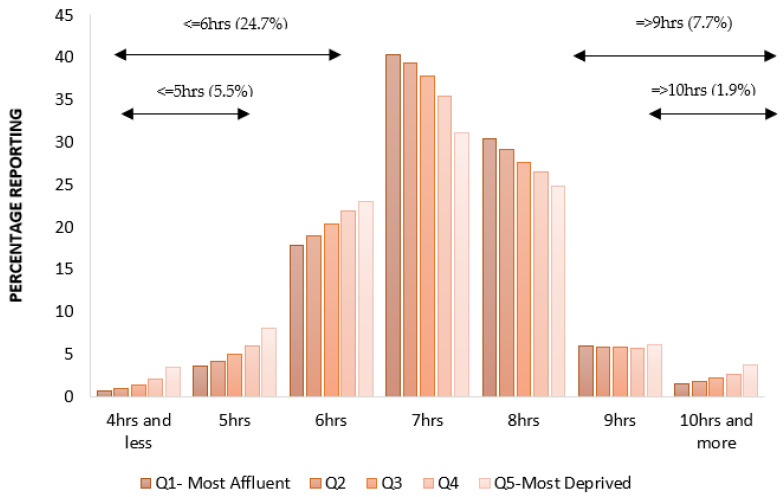
Percentage reporting different nightly sleep durations as a function of social deprivation.

**Figure 2:**
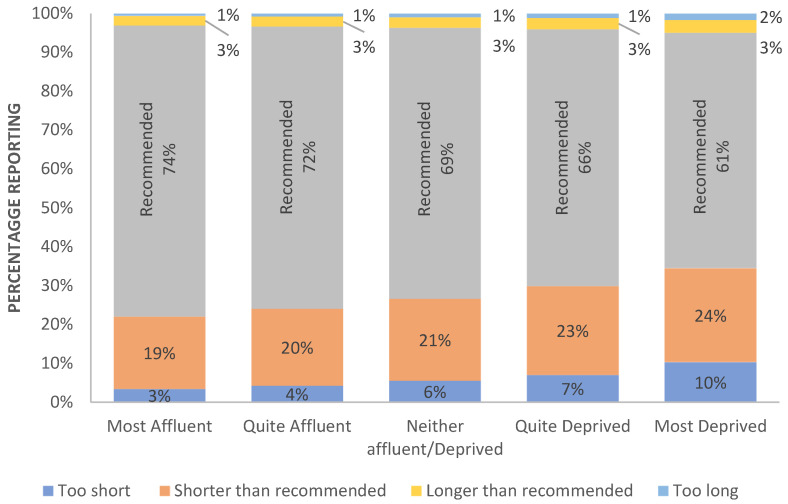
Percentage reporting recommended and less ideal nightly sleep durations as a function of social deprivation. For convenience, recommended sleep duration are labelled on the figure itself.

**Figure 3:**
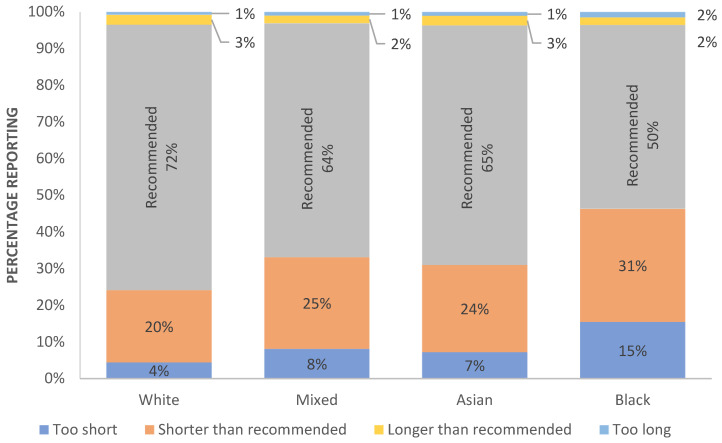
Percentage reporting recommended and less ideal nightly sleep durations as a function of ethnicity/race. For convenience, recommended sleep durations are labelled on the figure itself.

Figure 1 presents percentages of different sleep durations across domiciliary deprivation. Prevalence of longer- or shorter- durations are associated strongly with social deprivation (χ^2^_(16)_ = 5783.42, *p* < 0.0001; n = 497,672; ϕ = 0.108, Figure 2). When the sample size is corrected (i.e., ϕ /sqrt(df)), the effect of social deprivation on the adequacy of sleep duration is between a medium (0.075) and large (0.125) effect. Those living in more affluent areas are significantly more likely to report recommended durations; the percentage of those reporting shorter or longer sleep increases significantly with deprivation. Self-declared ethnicity collapsed into four overarching categories, affected the prevalence of reported sleep duration (χ^2^_(12)_= 3505.48, *p* < 0.0001; n = 489,880; ϕ = 0.085, small, 0.029 to medium, 0.087, see Figure 3), with White (72.4%) participants being more likely to report typical durations within the recommended range than those in Asian (65.4%), Mixed (63.8%) or Black (50.1%) groups. Incidence of extremely short and long durations was also influenced by ethnicity (χ^2^_(3)_ = 49.49, *p* < 0.0001; n = 464,592; ϕ = 0.043, small, 0.058), but although statistically reliable, the effect size is negligible.

### 2.2. Individual Sleep Problems

Sleep problems were worse among those living in more deprived areas. Domiciliary deprivation is associated with finding it harder to get up in the morning (χ^2^_(12)_ = 3128.36, *p* < 0.0001; n = 496,215; ϕ = 0.079, small, 0.029 to medium, 0.085), being more likely to nap (χ^2^_(8)_ = 2344.38, *p* < 0.0001; n = 499,994; ϕ = 0.068, small, 0.035) or dozing during the daytime (χ^2^_(8)_ = 389.33, *p* < 0.0001; n = 119,896; ϕ = 0.057, small, 0.035), and night-time wakefulness (χ^2^_(8)_ = 714.99, *p* < 0.0001; n = 119,896 ϕ= 0.038, small, 0.035). Reports of snoring also increase with social deprivation (χ^2^_(4)_ = 83.96, *p* < 0.0001; n = 464,592; ϕ = 0.013), but the effect size of that association is less than small (χ^2^_(crit, 4)_ <> 0.05).

Ethnicity also influenced the prevalence of sleep problems. White people (82.4%) considered it easier to get up in the morning compared with Black (74.4%), Asian (73.5%) or Mixed ethnicities (74.2%, χ^2^_(9)_ = 1061.647, *p* < 0.0001; n = 488,420; ϕ = 0.047, small, 0.033). More White (28.5%) or Mixed (29.2%) ethnicity participants reported ‘usually’ waking at night compared with those grouped as Black (20.2%) or Asian (22.1%; χ^2^_(6)_ = 520.148, *p* < 0.0001; n = 492,468; ϕ = 0.032, small, 0.041). Those who were Asian (41.1%) were more likely to snore than other ethnicities (White: 37.2%, Mixed: 34.0%, Black: 34.6%, χ^2^_(3)_ = 90.623, *p* < 0.0001; n = 457,996; ϕ = 0.014, small, 0.058). Daytime napping was less prevalent in White and Mixed ethnicities (Never/Rarely: 56.6%, 57.9% respectively) than those describing themselves as Asian or Black (48.7% 46.3% respectively; χ^2^_(6)_ = 590.983, *p* < 0.0001; n = 492,203; ϕ = 0.035, small, 0.041). White (11.5%) people were also less likely to report ‘often’ or ‘always’ dozing in the daytime than those of Black (15.5%), Asian (14.8%, Mixed 15.5% ethnicity (χ^2^_(6)_ = 78.905, *p* < 0.0001; n = 117,082; ϕ = 0.026, small, 0.041). Obviously, these self-reported problems may not be independent of each other, nor are their dependence on a range of other socio-demographic factors easily quantified.

The patterns of differences reported above raise the possibility that reports of particular sleep problems are not independent. Supplementary Materials Section S4 shows that this is the case—although generally small, all of the correlations are significantly related to each other (*p* < 0.0001). The exception is the ease of getting up in the morning is unrelated to snoring (*p* = 0.713). Otherwise, the correlations constitute a ‘positive manifold’, which would be indicative of a single underlying latent variable.

### 2.3. Problematic Sleep Index

To address these discussed above, we propose a pragmatic definition of good sleep comprising sleeping for the recommended duration, not waking in the night, but waking easily afterwards, not needing or having further sleep during the daytime, as well as the absence of snoring. This ‘Problematic Sleep Index’ combined all of these into a single measure, allowing us to quantify the influence on sleep quality of a wide range of participant characteristics. Supplementary Materials Section S5 further shows indices related to the reliability of this new measure. 

Linear regression identified the specific contributions of different demographic characteristics to the Problematic Sleep Index (Table 2). Results revealed a statistically significant association between the Problematic Sleep Index and a wide range of personal, societal and racial characteristics, *F*_(13, 338614)_ = 1536.592, *p* < 0.0001, *R^2^* = 0.056, _adj_*R^2^* = 0.056, Cohen’s *f^2^* = 0.0018. Better sleep is associated with being male, younger, affluent, educated to a degree level, living with others in the home you own and have lived in for some time, with a high income, multiple vehicles, and having a job for some time. While the overall variance accounted for adults is relatively small (6%), this must be set beside the myriad of factors which underlie variability in such a large sample. The tabulated standardised beta weights indicate the independent explanatory contribution of each variable and starkly illustrate the social inequalities underlying sleeping well. Being employed or retired is associated with better sleep; being unable to work or unemployed is not. Men sleep better, but the influence of biological sex is only of a similar extent to that of household income. Domiciliary deprivation exerts more influence on sleep quality than age or the type of property in which participants lived. Ethnicity, particularly being White, is associated with better sleep.

**Figure 4:**
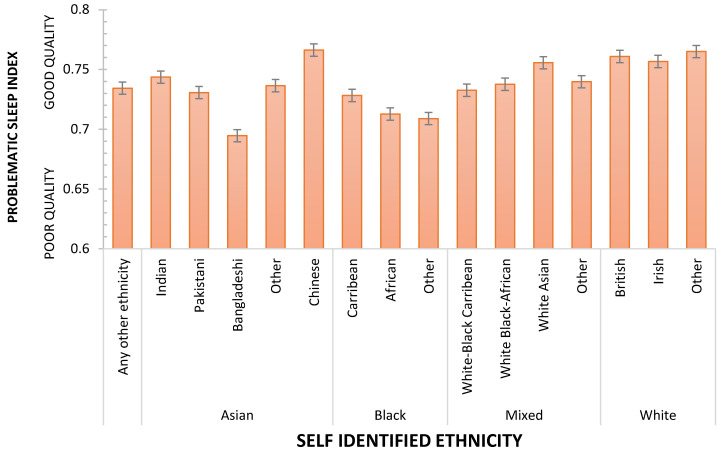
Problematic Sleep as a function of ethnicity/race. Dashed line shows arithmetic mean, error bars are SE.

**Figure 5:**
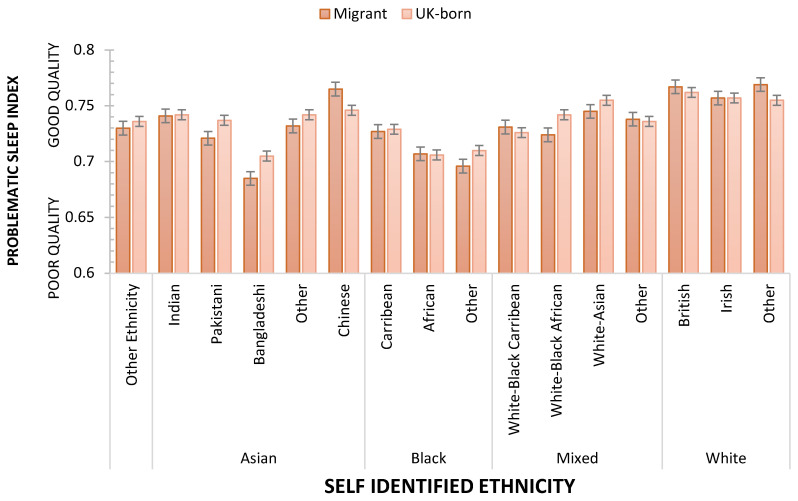
Problematic Sleep as a function of Ethnicity/Race and Nativity. Dashed line shows arithmetic mean, error bars are SE.

**Figure 6:**
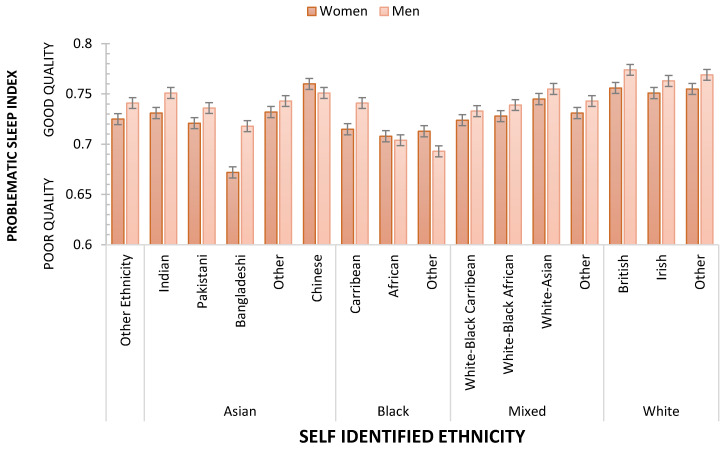
Problematic Sleep as a function of Ethnicity/Race and Biological Sex. Dashed line shows arithmetic mean error bars are SE.

It is striking that age and sex, characteristics we as sleep researchers consider of particular importance, are only of similar importance to personal wealth and the level of deprivation of the communities in which we live. Consistent with other studies, nativity also influences sleep, but these results show that the benefit of being born in the country of domicile is weaker than other socio-demographic characteristics mentioned above. UKB’s size and diversity allow the effects of nativity and ethnicity to be explored further. An ANCOVA, controlling for age, was used to assess the effects of ethnicity, nativity, and sex on Problematic Sleep. Main effects were observed for ethnicity (F_(15, 409041)_ = 59.261 *p* < 0.001; η_p_^2^ = 0.0021687), sex (F_(1, 409041)_ = 12.640; *p* < 0.001; η_p_^2^ = 0.000031; Men sleep better than Women) but not nativity (F < 1; *p* > 0.347). Figure 4 illustrates the differences between ethnicities in terms of Problematic Sleep. Table 3 summarises the outcome of FDR-controlled contrasts between all ethnicities, showing that the sleep of White respondents was better than all other ethnic groups, and, for example, Chinese participants had fewer sleep problems than each of the other Asian ethnicities. Two interactions indicated that ethnicity’s influence was separately modified by nativity (F_(15, 409041)_ = 3.445730 *p* < 0.001; η_p_^2^ = 0.000126, Figure 5) and sex (F_(15, 409041)_ = 2.074; *p* < 0.01; η_p_^2^ = 0.000076, Figure 6). While the main effect of sex shows that overall, men sleep better than women, this is not consistent across all ethnicities. Specifically, Chinese and Black women who are neither African nor Caribbean sleep better than their male counterparts. The sleep quality of Black African women and men is not statistically different.

FDR corrected Welch contrasts show a Problematic Sleep advantage for some UK-born ethnicities, notably Asian (Indian, Pakistani, Other), Black (Caribbean), Mixed (White-Black African, White-Asian) ethnicities; but sleep is better for some migrant groups, notably Asian (Chinese), Black (African), Mixed (White-Black Caribbean, Other) and White (British, Irish, Other), than for those of the same ethnicity born in the UK. Further exploration of these equivocal effects of nativity on sleep is beyond the scope of this paper, but their complexity should not be understated (e.g., ethnicities differ in terms of age at migration, years domiciled in the UK, and year of migration), and it is notable that in this UK sample nativity was the weakest of all affluence–ethnicity characteristics which affected sleep quality.

## 3. Method

This cross-sectional study is based on data from the UKB study; its design and methods have been reported in detail previously [28]. Participants provided written informed consent for data collection, data (re-)analysis, and record linkage (NHS National Research Ethics Service 16/NW/0274; 2011, 2016, 2021).

### 3.1. Study Population

Approximately 500,000 adults aged 40–69 years, registered with the UK National Health Service and living <25 miles of widely dispersed assessment centres, accepted one of 9 m invitations sent between 2006 and 2010. Some 96% of the data reported were collected between July 2007 and June 2010, with equal improvement across months of each year, except for December, which was about 50% of that achieved in other months.

### 3.2. Data Sources and Management

Data were collected on socio-demographic, lifestyle, and health behaviour variables. Here we report analyses based on age and sex, self-identified ethnicity, education, accommodation, wealth, and employment. In UKB, Participants’ post-codes were used to generate Townsend Deprivation Index (TDI) for the areas in which they lived by combining four questions from the UK2011 census [Unemployed economically active people (%); Overcrowded households (i.e., number of Occupants > Rooms, %); Households not owning car/van; households renting/living rent free]. Townsend data are used below, re-scored to reflect the quintile score of that post-code in terms of the affluence of the UK population [29].

Participants reported their sleep by rating rated how often they experienced various sleep problems. ‘Do not know’/‘prefer not to answer’ responses were treated as missing values, and some responses were re-coded, as detailed in Supplementary Materials Sections S1 and S2.

### 3.3. Statistical Analyses

A Problematic Sleep Index (PSI) based on five sleep challenges was calculated for each participant. This comprised evidence of (i) Inadvisable sleep duration, where sleep length was re-coded into 5 age-based levels (Much-/Lower-than recommended, Recommended, Higher/Much Higher than recommended, scored as 2, 1, 0, 1, 2) [14], (ii) Snoring (Yes (1)/No (0)), (iii) Sleeplessness: Never/rarely (0), Sometimes (1), Usually (2), (iv) Difficulty waking independent of Chronotype was estimated using residuals from (ease of) Getting up in the morning (Not at all, Not very, Fairly, Very; scored as 4, 3, 2, 1) following regression of Chronotype (Definitely morning, more morning than evening, more evening than morning, Definitely evening), (v) Daytime sleepiness combined Napping during day (Never/rarely (0), Sometimes (1), Usually (2)), and likelihood of unintended dozing (Never/rarely (0), Sometimes (1), Often (2)), using Principal Components Analysis (PCA) to derive a single factor. These five components were combined into a component using PCA, factor scores from which were rescaled such that ‘1′) represented the best possible sleep: sleeping for the recommended duration, waking easily following that sleep, not sleeping during the day, falling asleep easily and or not waking, or snoring, during sleep. (Further details of the central tendencies, dispersion and PCA solutions are provided in Supplementary Materials Section S3).

Various approaches were adopted to counter the downsides of analysing very large samples. With very large sample sizes, there is a need to avoid potentially spurious effects with conventional α-levels of 0.05. Standardising the α-level for a sample of 50,000 would suggest a cut-off of 0.002 and for 500,000 of 0.0007 [30]. Accordingly, we regard *p* < 0.001 as a conservative benchmark for significance throughout. For larger contingency table analyses, we apply the recommended effect size correction (i.e., ϕ/sqrt(df)) [31], which substantially reduces Cohen’s conventional 0.1, 0.3 and 0.5 as guidelines for small, medium, and large ϕ effects [32]. We also adapted a heuristic to assess effect-size importance of small effects by comparing relevant effect sizes with those from manipulations generally considered to be important (i.e., comparing effect sizes with those from, for example, sex or age differences) [33]. Where contrasts were carried out between sub-samples differing in size, Welch tests, which are robust against unequal variances and substantial sample size differences, were used. Finally, to address issues arising from multiplicity and false discovery, all post hoc comparisons were FDR corrected [34].

All analyses were performed in IBM SPSS Statistics 26.0.0.0.

## 4. Discussion

Higher or lower than age-appropriate sleep duration, waking during the night, the likelihood of sleeping during the day, difficulty getting up in the morning and snoring are all affected by the ethnicity of the respondent and the extent of social deprivation where they live. Combining these sleep characteristics into a single measure, the Problematic Sleep Index, we show that sleep is worse in those who are not White and/or live in less affluent areas. Moreover, the sample size is sufficiently large to demonstrate that both social deprivation and ethnicity affect sleep quality independently of age, sex, personal wealth, employment, and education. While the effect sizes are small, unsurprising given the very large sample size, all are greater than age, which is typically seen as exerting the most profound influence on sleep.

One-third of respondents reported sleeping for less (24.7%) or more (7.7%) than is recommended. This is concerning since the prevalence of major diseases is greater among short- and long-sleepers. Given the separate effects of being White and/or affluent, these data imply that sleep-related health conditions may also differ in prevalence according to social deprivation and ethnicity. There is increasing evidence that inadequate sleep compromises immune reactions and that good sleep has a protective effect [35]. Our findings suggest that these benefits and challenges are disproportionally distributed across the disadvantaged and those who are not White. Even within these other ethnicities, despite statistically controlling effects of the deprivation of the area in which participants lived, personal wealth, age, education and sex, there are notable differences (e.g., Bangladeshi vs. other Asians, Black Caribbeans vs. Africans) that warrant further attention. Men did not sleep better than women across all ethnicities (i.e., Black African or Mixed-race men and women similar). In a study of four South African racial groups, Black African women slept more than men, White African women slept less, and male and female Multi-Ancestry and Indian/Asian African women reported sleeping similar amounts [36]. That study, among many others, uses relatively arbitrary, age-insensitive criteria for adequacy of sleep duration. We see it as a particular strength of the current study that we rely on age-corrected expert consensus and encourage other authors to do likewise.

Data from the Ontario study cited above [26] show that White participants slept more than other ethnicities, even when their statistical model took years of residence in Canada into account. They do not report whether residency covaried significantly with sleep duration. In multicultural societies such as the UK, duration of residency, and acculturation, may have a substantial influence on the reporting of sleep and health complaints [37] and perhaps in preparedness/availability to take part in longitudinal studies. That aside, consistent with the Canadian study [26], but unlike data from South Africa [36], we show that White participants reported longer sleep than other groups. Nativity, on the other hand, has a relatively slight influence on our study.

Our results show that domiciliary deprivation was associated with more problematic sleep. As mentioned earlier, our index of social deprivation is derived from census information and is used widely at the governmental level for regional contrasts. This effect of the domiciliary area is separate from that of personal or household wealth and thus implies some wider environmental influences may underlie the disparity in sleep quality, such as noise, pollution, neighbourhood safety, green space access, etc. [38,39]. This, as well as the links between sleep quality and ethnicity, may have important implications for how sleep challenges might be ameliorated, with the potential for community-led interventions tailored to that community’s identity rather than a relatively mass appeal to undifferentiated individuals.

### Limitations

While the sample analysed is large, the effects reported account for very small amounts of variance. We have attempted to address this in a variety of ways by reporting effect size criteria corrected for sample sizes but also by considering the amount of influence variables have in relation to the influence of variables typically regarded as important by the sleep research community [32].

Our results come exclusively from self-reports, and thus the Problematic Sleep Index may lack reliability and validity. We have addressed this by calculating the index on different parts of the sample and showing these estimated generalise to other samples. The Problematic Sleep Index has recently been shown to be associated with systemic chronic inflammation (SCI, i.e., platelet counts, C-reactive protein levels, and neutrophil-to-lymphocyte ratios) [40].

The data reflect reports made almost a decade ago and obscure possible differences between work-day and rest-day sleep. Furthermore, what is asked about sleep in the whole sample is also rather rudimentary and incomplete. UKB sleep questions vary from requiring reports of specific numbers of hours slept, completion of three- and four-item scales and even binary reports, and we acknowledge here the psychometric shortcomings of this [41,42]. Similarly, it is unfortunate that UKB did use questions which mapped to clinical criteria for insomnia (e.g., difficulty initiating sleep, waking early without being able to return to sleep, etc.) or hypersomnia. One of the motivations for developing the Problematic Sleep Index was to attempt to minimise some of these shortcomings, and it is noteworthy that the effects of domiciliary disadvantage and ethnicity found with this new index are echoed in responses on the original, simpler scales. Finally, in terms of measurement limitations, it might be objected that our deliberate creation of a single index obscures the multifaceted nature of sleep complaints and disorders. While this is undeniably true for disorders, we suggest that at their heart, the sleep problems people complain about share a dissatisfaction that the sleep obtained is worse than the sleep desired. As such, we conceptualise Problematic Sleep as an “index” rather than as a “construct” in the strict psychometric sense [43].

Given the clear effect of social deprivation, it is also unfortunate that there is less information available about the physical and social characteristics of the environments in which respondents live. These weaknesses must be set alongside the sheer extent of the UK Biobank dataset, with the consequent scope for establishing the independence of effects which smaller studies confound or cannot address. That acknowledged, while the UK Biobank is a very large dataset drawn from across the United Kingdom, it is not ‘representative’ of the UK population in the strict sense. According to a very recent assessment which contrasted UKB demographics with census data, “Biobank participants were more likely to be older, to be female, and to live in less socioeconomically deprived areas than nonparticipants. Compared with the general population, participants were less likely to be obese, to smoke, and to drink alcohol on a daily basis and had fewer self-reported health conditions” [44]. While this is an important caveat, its implications are that sleep quality may well be worse than we report it to be.

## 5. Conclusions

The analyses reported above show that Problematic Sleep is unequally distributed across the UK population, with the possibility that these inequalities increase the health risks some groups face. Since there is abundant evidence that sleep can be improved, it may be that specifically addressing sleep issues among particular social groups in their local areas can enhance health and quality of life. In particular, we suggest that the Problematic Sleep Index, introduced above, can be easily used by those involved in community-led health care to identify individuals and families living in locations within particular postal areas where support for specific sleep problems can be targeted very precisely. Even without doing so, we believe the analyses reported above might encourage general practitioners and others engaged with integrative care to consider disparities in sleep quality which relate to ethnicity and social deprivation.

## Figures and Tables

**Table 1:** Sample characteristics in relation to Ethnicity/Race, Social Deprivation and Sex.

ETHNICITY/RACE	N	Most Affluent	Quite Affluent	Neither Affluent/Deprived	Quite Deprived	Most Deprived
**OTHER ETHNICITY/RACE**	4542	19 (58)	15 (61)	19 (57)	24 (59)	23 (50)
**ASIAN**						
Indian	5944	25 (49)	23 (49)	26 (51)	20 (48)	6 (46)
Pakistani	1835	17 (41)	16 (44)	19 (39)	32 (38)	15 (35)
Bangladeshi	236	9 (38)	14 (38)	16 (45)	22 (25)	39 (25)
Other	1851	21 (53)	17 (48)	25 (43)	22 (44)	15 (42)
Chinese	1569	36 (62)	20 (67)	21 (62)	15 (63)	9 (59)
**BLACK**						
Caribbean	4511	11 (60)	15 (61)	21 (64)	29 (66)	24 (63)
African	3380	7 (58)	9 (48)	15 (51)	26 (51)	44 (48)
Other	150	8 (58)	15 (59)	20 (77)	25 (76)	33 (57)
**MIXED**						
White-Black Caribbean	620	22 (56)	18 (72)	19 (66)	25 (62)	17 (60)
White-Black African	425	19 (62)	14 (57)	21 (73)	25 (72)	21 (80)
White Asian	831	34 (61)	20 (56)	19 (60)	19 (55)	9 (55)
Other	1081	27 (66)	19 (65)	18 (65)	21 (61)	15 (57)
**WHITE**						
British	44,2059	48 (54)	22 (55)	14 (55)	11 (54)	4 (49)
Irish	13,195	36 (54)	22 (54)	17 (53)	15 (49)	9 (45)
Other	16,879	32 (61)	21 (62)	19 (61)	18 (61)	10 (57)

Note: Rows show Affluence frequencies for each ethnicity/race, with percent of women in each subsample in parentheses.

**Table 2:** Predictors of Problematic Sleep.

	B	SE	β	95% CI	t	*p*	Cohen’s f ^2^
(Constant)	0.8072	0.0020			409.3759	0.0000	
Sex (0, F; 1, M)	0.0164	0.0004	0.0756	0.8034, 0.8111	44.9533	0.0000	0.00566
Age (40,45, 50, 55, 60, 65, 70+, 1–7)	−0.0023	0.0001	−0.0347	0.0157, 0.0171	−17.0040	<0.001	0.00078
Qualifications (Degree to none)	−0.0020	0.0001	−0.0346	−0.0026, −0.0020	−19.4605	<0.001	0.00103
Deprivation (Most to Least Affluent, 1–5)	−0.0046	0.0002	−0.0537	−0.0022, −0.0018	−27.2523	<0.001	0.00212
Ethnicity/Race (White, 1; Mixed, 2; Asian, 3; Black, 4)	−0.0087	0.0006	−0.0266	−0.0050, −0.0043	−13.7062	<0.001	0.00053
Nativity (1-UK born, 0: Migrant)	−0.0015	0.0007	−0.0039	−0.0099, −0.0074	−1.9689	0.0490	0.00001
Accommodation (House,1; Flat, 2; Mobile,3)	−0.0008	0.0006	−0.0025	−0.0029, 0.0000	−1.2828	0.1996	0.00000
Owning, renting, living rent free (1,2,3)	−0.0148	0.0006	−0.0461	−0.0020, 0.0004	−23.6342	<0.001	0.00152
Number in household	−0.0002	0.0002	−0.0021	−0.0160, −0.0135	−1.0372	0.2997	0.00003
Number in household years at address	−0.0004	0.0001	−0.0058	−0.0006, 0.0002	−3.1297	0.0017	0.00000
Total household income	0.0069	0.0002	0.0765	−0.0007, −0.0002	34.9370	<0.001	0.00338
Number of vehicles	0.0005	0.0003	0.0042	0.0065, 0.0073	1.9813	0.0476	0.00001
Paid job, 1; Retired, 2; Home/Carer, 3; Voluntary, 4; Unable, 5; Unemployed, 6	−0.0123	0.0002	−0.1169	0.0000, 0.0010	−64.7604	0.0000	0.01180

**Table 3:** Ethnicity/Race differences in Problematic Sleep.

		ASIAN					BLACK			MIXED				WHITE		
	Other Ethnicity	Indian	Pakistani	Bangladeshi	Other	Chinese	Caribbean	African	Other	White-Caribbean	White-Black African	White Asian	Other	British	Irish	Other
**OTHER ETHNICITY/RACE**						*p* *		N *						*p* *	*p* *	*p* *
**ASIAN**																
Indian							N *	N *						*p* *	*p* *	*p* *
Pakistani						*p* *		N *						*p* *	*p* *	*p* *
Bangladeshi														*p* *		*p* *
Other								N *						*p* *		*p* *
Chinese	N *		N *				N *	N *	N *							
**BLACK**																
Caribbean		*p* *				*p* *		N *				*p* *		*p* *	*p* *	*p* *
African	*p* *	*p* *	*p* *		*p* *	*p* *	*p* *				*p* *	*p* *	*p* *	*p* *	*p* *	*p* *
Other						*p* *						*p* *		*p* *	*p* *	*p* *
**MIXED**																
White-Caribbean														*p* *	*p* *	*p* *
White-Black African								N *						*p* *		*p* *
White Asian							N *	N *	N *							
Other								N *						*p* *	*p* *	*p* *
**WHITE**																
British	N *	N *	N *	N *	N *		N *	N *	N *	N *	N *		N *		N *	
Irish	N *	N *	N *				N *	N *	N *	N *			N *	*p* *		*p* *
Other	N *	N *	N *	N *	N *		N *	N *	N *	N *	N *		N *		N *	

Note: The table shows the direction and FDR corrected significance level (*p* * (positive)/N * (negative), *p* = 0.001) for all statistically significant, differences in Problematic Sleep for each ethnic group, such that the column-row contrast—POS when White Irish sleep is better than Pakistani sleep, and NEG when Pakistani (column) sleep is worse than Irish (row) sleep. All comparisons based on estimated means adjusted to remove effects of age.

## Data Availability

All relevant data are within the paper, all other data available by application from UK Biobank.

## Supplementary Materials

The following supporting information can be downloaded at: https://www.mdpi.com/article/10.3390/clockssleep5030030/s1, Table S1: Response Options, Recoding and Descriptive Statistics; Section S1: UK Biobank Sociodemographic Indices Relevant to Problematic Sleep Study; Section S2: Sleep Variables; Section S3: Principal Components Analysis/Principal Axis Factoring; Section S4: Intercorrelation of Sleep Problem Reports; Section S5: Problematical Sleep Index: Reliability.
