# Supplementary material for: Social Deprivation and Ethnicity Are Associated with More Problematic Sleep in Middle-Aged and Older Adults
Source: Clocks Sleep. 2023 Aug 7;5(3):399–413. doi: 10.3390/clockssleep5030030 (PMC10443366; doi:10.3390/clockssleep5030030)
Supplement: Supplementary file 1 [file clockssleep-05-00030-s001.zip › clockssleep-2457205-supplementary.pdf]

1. UK Biobank Sociodemographic Indices Relevant to Problematic Sleep Study

Self-Identified Ethnicity: Response Options

White: British, Irish, Any other white background; **Mixed**: White and Black Caribbean, White and Black African, White and Asian, Any other mixed background; **Asian or Asian British**: Indian, Pakistani, Bangladeshi, Chinese, Any other Asian background; **Black or Black British**: Caribbean, African, Any other Black background; **Other ethnic group**

Education

1 College or University degree
2 A levels/AS levels or equivalent
3 O levels/GCSEs or equivalent
4 CSEs (Certificate of Secondary Education) or equivalent
5 NVQ or HND or HNC or equivalent
6 Other professional qualifications e.g.: nursing, teaching

Employment

1 In paid employment or self-employed
2 Retired
3 Looking after home and/or family
4 Unable to work because of sickness or disability
5 Unemployed
6 Doing unpaid or voluntary work
7 Full or part-time student

Accommodation

1 A house or bungalow
2 A flat, maisonette or apartment
3 Mobile or temporary structure (i.e., caravan)
4 Sheltered accommodation
5 Care home

Home Ownership

1 Own outright (by you or someone in your household)
2 Own with a mortgage
3 Rent - from local authority, local council, housing association
4 Rent - from private landlord or letting agency
5 Pay part rent and part mortgage (shared ownership)
6 Live in accommodation rent free

Household income

1 Less than 18,000
2 18,000 to 30,999
3 31,000 to 51,999
4 52,000 to 100,000
5 Greater than 100,000

NB In 2010 Median individual earnings across the UK was £25,881. In 2022 this was £33,000

2. Sleep Variables:

Table S1 Response Options, Recoding and Descriptive Statistics

	N, (% sample)	Mean, SE	Median, Range	Skewness, SE	Kurtosis, SE
**Getting up in the morning**					
not at all easy	19760, (4)	3.038,0.0026	3,1-4	-0.649,0.008	-0.062,0.016
not very easy	69938, (14.1)				
fairly easy	246646, (49.6)				
very easy	160487, (32.3)				
**Chronotype Morning to Evening**					
definitely a morning person	120364, (27.1)	2.170,0.003	2,1-4	0.303,0.008	-0.912,0.016
more a morning than evening person	157377, (35.4)				
more an evening than a morning person	126333, (28.4)				
definitely an evening person	40110, (9)				
**Daytime dozing**					
Sometimes	105969, (88.3)	1.115,0.001	1,1-3	2.432,0.008	4.006, 0.106
Often	14052(11.7)				
Always	43, (0)				
**Nap during day**					
Rarely/Never	281069, (56.1)	1.834,0.001	2,1-3	0.064,0.008	-0.332,0.016
Sometimes	192655, (38.5)				
Usually	26886, (5.4)				
**Sleeplessness insomnia**					
Never/Rarely	105969, (88.3)	1.115,0.001	1,1-3	2.432,0.008	4.006,0.016
Sometimes	14052, (11.7)				
Usually	43, (0)				
**Snoring**					
Yes	173360, (37.2)	1.555,0.001	2,1-2	-0.225,0.008	-1.949,0.016
No	292092, (62.8)				
**Recommended Sleep durations (recoded as 0, recommended, 1 Perhaps appropriate, 2, not recommended**					
		1.424,0.001	1,1-0.630	1.203,0.008	0.309,0.015
**Daytime sleepiness (factor score from PCA reduction of Nap during day,**					
		-0.004,0.003	-0.058,-1.125	1.242,0.008	1.636,0.015
**Ease of rising controlled for Morningness-Eveningness**					
		-0.078,0.002	-0.175,-2.567	-0.57,0.008	0.342,0.015

3. Principal Components Analysis/ Principal Axis Factoring

Variables entered: Age-corrected Recommended Sleep durations, Sleeplessness insomnia, Snoring, Daytime sleepiness, Ease of rising (see Table S1)

Total Variance Explained

Component	Initial Eigenvalues		
	Total	% of Variance	Cumulative %
1	1.312	26.241	26.241
2	1.002	20.64	46.881
3	0.952	19.031	65.912
4	0.886	17.714	83.631
5	0.818	16.369	100

Scree test indicated single factor solution

Component	Initial Eigenvalues		
	Total	% of Variance	Cumulative %
1	1.324	26.473	26.473
2	0.995	19.602	46.075
3	0.943	18.85	65.926
4	0.917	18.347	84.273
5	0.768	15.727	100

Extraction Method: Principal Axis Factoring.

Scree test indicated single factor solution

Factor scores from the single component/factor created using PCA/PAF are highly correlated $r_{(410227)}$= 0.961606.

4. **Intercorrelation of Sleep Problem Reports**

	1	2	3	4	5	6	7
**1. Sleep duration** (1-23 hrs)	**1**	493843	442061	497534	497890	118820	462919
**2. Ease of getting up** (not at all/not very/fairly/very easy)	-0.023**	**1**	443767	496096	496455	118922	461351
**3. Chronotype** (definitely morning, more morning, more evening, definitely evening)	0.029**	-0.468**	**1**	443755	443942	106639	413771
**4. Nap during day** (never, rarely/sometimes/usually)	0.124**	-0.017**	-0.023**	**1**	500258	119748	464855
**5. Sleeplessness/insomnia** (never, rarely/sometimes/usually)	-0.233**	-0.116**	0.006**	0.057**	**1**	119935	465163
**6. Daytime dozing** (sometimes/often/always)	0.017**	-0.090**	0.011**	0.246**	0.087**	**1**	110638
**7. Snoring** (yes/no)	-0.022**	0.001	-0.014**	-0.072**	0.032**	-0.039**	**1**

** Pearson correlation is significant at the 0.001 level (2-tailed). Sample size for each correlation above diagonal.

5. Problematical Sleep Index: Reliability

To assess the reliability of this new index, these calculations were repeated for two randomly selected cohorts from the whole sample, the Problematic Sleep Index was not statistically different for the first (N= 48,618; 0.7194$\pm$.1271) and second (N= 48,815; .7190$\pm$.1276) of these cohorts ($t_{(97,750)}$= 0.486, p>.63). Separate linear regressions were calculated for each cohort, the r-square values in each case showed that the raw responses constituted most of the variance in each index (both 92%). First cohort regression equation was used to generate predicted scores for the second cohort and vice versa. Correlations between these predicted and actual values for each cohort demonstrated that these regression weighs generalised well to ‘new’ samples ($r_{(48,599)}$=0.709; $r_{(48,795)}$=0.704; both p<.00001).
